# Tracing defaulters in HIV prevention of mother-to-child transmission programmes through community health workers: results from a rural setting in Zimbabwe

**DOI:** 10.7448/IAS.18.1.20022

**Published:** 2015-10-12

**Authors:** Florian Vogt, Cecilia Ferreyra, Andrea Bernasconi, Lewis Ncube, Fabian Taziwa, Winnie Marange, David Wachi, Heiko Becher

**Affiliations:** 1 Operational Centre Barcelona, Médecins Sans Frontières/Doctors Without Borders, Barcelona, Spain; 2 Tsholotsho Project, Médecins Sans Frontières/Doctors Without Borders, Tsholotsho, Zimbabwe; 3 Zimbabwe Mission, Médecins Sans Frontières/Doctors Without Borders, Harare, Zimbabwe; 4 Tsholotsho District Medical Office, Ministry of Health and Child Welfare, Tsholotsho, Zimbabwe; 5 Department of Medical Biometry and Epidemiology, University Medical Centre Hamburg-Eppendorf, Hamburg, Germany

**Keywords:** HIV, prevention of mother-to-child transmission, community health workers, defaulter tracing, retention in care, vertical transmission, Zimbabwe, Médecins Sans Frontières/Doctors Without Borders

## Abstract

**Introduction:**

High retention in care is paramount to reduce vertical human immunodeficiency virus (HIV) infections in prevention of mother-to-child transmission (PMTCT) programmes but remains low in many sub-Saharan African countries. We aimed to assess the effects of community health worker–based defaulter tracing (CHW-DT) on retention in care and mother-to-child HIV transmission, an innovative approach that has not been evaluated to date.

**Methods:**

We analyzed patient records of 1878 HIV-positive pregnant women and their newborns in a rural PMTCT programme in the Tsholotsho district of Zimbabwe between 2010 and 2013 in a retrospective cohort study. Using binomial regression, we compared vertical HIV transmission rates at six weeks post-partum, and retention rates during the perinatal PMTCT period (at delivery, nevirapine [NVP] initiation at three days post-partum, cotrimoxazole (CTX) initiation at six weeks post-partum, and HIV testing at six weeks post-partum) before and after the introduction of CHW-DT in the project.

**Results:**

Median maternal age was 27 years (inter-quartile range [IQR] 23 to 32) and median CD4 count was 394 cells/µL^3^ (IQR 257 to 563). The covariate-adjusted rate ratio (aRR) for perinatal HIV transmission was 0.72 (95% confidence intervals [95% CI] 0.27 to 1.96, *p*=0.504), comparing patient outcomes after and before the intervention. Among fully retained patients, 11 (1.9%) newborns tested HIV positive. ARRs for retention in care were 1.01 (95% CI 0.96 to 1.06, *p*=0.730) at delivery; 1.35 (95% CI 1.28 to 1.42, *p*<0.001) at NVP initiation; 1.78 (95% CI 1.58 to 2.01, *p*<0.001) at CTX initiation; and 2.54 (95% CI 2.20 to 2.93, *p*<0.001) at infant HIV testing. Cumulative retention after and before the intervention was 496 (85.7%) and 1083 (87.3%) until delivery; 480 (82.9%) and 1005 (81.0%) until NVP initiation; 303 (52.3%) and 517 (41.7%) until CTX initiation; 272 (47.0%) and 427 (34.4%) until infant HIV testing; and 172 (29.7%) and 405 (32.6%) until HIV test result collection.

**Conclusions:**

The CHW-DT intervention did not reduce perinatal HIV transmission significantly. Retention improved moderately during the post-natal period, but cumulative retention decreased rapidly even after the intervention. We showed that transmission in resource-limited settings can be as low as in resource-rich countries if patients are fully retained in care. This requires structural changes to the regular PMTCT services, in which community health workers can, at best, play a complementary role.

## Introduction

Perinatal transmission of human immunodeficiency virus (HIV) infection from mother-to-child is the main mode of HIV acquisition in children [1–3]. The risk of transmission is 15 to 40% in the absence of any medical intervention but can be reduced to 1 to 5% through antiretroviral therapy (ART) during pregnancy, delivery and breastfeeding [2–11]. As a consequence, prevention of mother-to-child transmission (PMTCT) programmes have become part of many HIV programmes in sub-Saharan Africa (SSA) [1, 7, 12–16].

In most of these settings, HIV-positive pregnant women who meet the ART eligibility criteria based on cluster of differentiation Type 4 lymphocyte (CD4) cell count and World Health Organization (WHO) clinical staging receive lifelong ART. For those women not eligible for treatment, WHO recommended until recently two prophylactic options: Option A or Option B [17, 18]. In both options, the intention of ART provision is prophylactic and ends with the cessation of breastfeeding. Latest WHO recommendations abandoned Option A in favour of Option B+, which means the initiation of lifelong ART for all HIV-positive pregnant women regardless of immunological or clinical status [19]. WHO guidelines promote HIV testing in all children born to HIV-positive mothers at four to six weeks post-partum using Deoxyribonucleic Acid detecting Polymerase Chain Reaction (DNA-PCR) technology [19, 20]. Due to the high risk for opportunistic infections, WHO also recommends cotrimoxazole (CTX) prophylaxis to prevent *Pneumocystis carinii* pneumonia for all infants of HIV-positive mothers starting at six weeks post-partum [21–23].

The sequence of diagnostic and treatment steps in PMTCT programmes defines a continuum of care, often referred to as “PMTCT cascade” [15, 18, 20, 24–31]. Modern PMTCT medication has proven to be highly efficacious and could lead to virtual elimination of paediatric HIV if universally implemented [15, 24, 32, 33]. This, however, would require high coverage and retention levels with timely service uptake at all recommended steps [25, 33–37]. While some SSA countries, such as Botswana, Namibia or South Africa, demonstrate that universal PMTCT coverage is feasible in low-resource settings, most SSA countries show major gaps [7, 10, 14, 16, 24, 35]. Delayed service uptake and high loss to follow-up (LTFU) along the PMTCT cascade is common across large parts of SSA [14, 28, 32, 34, 38–46].

Community health workers (CHWs) are defined as lay personnel without formal medical education who are recruited amongst the communities from which patients arise [47–56]. The benefits of using CHWs to trace defaulting patients have been shown in adult HIV programmes [57–59]. Most PMTCT programmes, however, still lack community involvement [15]. The few PMTCT programmes that do have a CHW component either use CHWs for tasks other than defaulter tracing [26, 38, 60–75] or are poorly monitored so that the effects of CHWs on programme outcomes remain unclear [24, 33, 35, 49, 76]. Evidence from community health worker–based defaulter tracing (CHW-DT) in PMTCT programmes involving household visits to defaulting patients is particularly scarce [15, 47–49].

To close this gap, we aimed at quantifying the effects of CHW-DT on vertical HIV transmission and retention in care in a rural resource-limited setting. We used routine programme data from HIV-positive pregnant women enrolled into a Doctors Without Borders (Médecins Sans Frontières, MSF) PMTCT project in the Tsholotsho district of Zimbabwe between February 2010 and March 2013 for a retrospective cohort analysis comparing outcomes before and after the introduction of CHW-DT.

## Methods

### Setting

Tsholotsho district is an arid, impoverished, rural district in south-western Zimbabwe with a population of approximately 115,000 [77]. Subsistence farming of drought-resistant crops and small-scale livestock herding are the main pillars of the local economy [78]. HIV prevalence is estimated at 18.3% among the general adult population, and at 20.2% among females of reproductive age [79].

In collaboration with the Ministry of Health and Child Welfare (MoHCW), MSF maintained an HIV/AIDS programme in Tsholotsho district between 2004 and 2014. A PMTCT component was introduced at Tsholotsho district hospital in 2006. In 2009, MSF started decentralizing PMTCT services to 14 health facilities. Two of these sites were located in a semi-rural setting within the district's principal settlement with relatively large numbers of patients compared with the 12 other sites. These, in contrast, were located remotely in very rural areas spread across the district.

At all facilities, pregnant mothers were encouraged to be tested for HIV during ante-natal care (ANC) visits. They were offered lifelong treatment for their own health if found to be positive and if they met ART eligibility criteria of having a CD4 count ≤350 cells/µL blood or being diagnosed with WHO Stage 3 or 4 disease. If they did not meet these criteria, Option A for prophylaxis was offered according to national guidelines at that time [80]. Between 2009 and 2013, treatment recommendations evolved from Option A towards B+ internationally [17–20, 81] but Option A remained in effect in Zimbabwe until the end of 2013 [82–84]. Consequently, in this setting, PMTCT was offered according to WHO Option A throughout the study period.

### Intervention

A CHW-DT system was introduced in the project in April 2012. Before that date, no defaulter tracing was carried out. After April 2012, all defaulting pregnant mothers and their newborns were traced in a standardized way. Volunteers from communities within the facilities catchment areas were recruited as CHWs to carry out home visits to defaulting patients. All CHWs received training in tracing and defaulter counselling techniques. In addition, educational workshops were organized for CHWs to strengthen their knowledge and promotion of basic health messages. These focused in particular on the importance of ante- and peri-natal PMTCT services as well as on infant vaccination. CHWs were not formally employed by the MSF project and did not receive financial remuneration or incentives for conducting the tracing. However, reimbursement of travel expenses and meals were provided when CHWs attended workshops outside their area of residence.

In the Tsholotsho project, a PMTCT defaulter was defined as: (1) a pregnant mother missing her scheduled ANC visit by more than two weeks; (2) a pregnant mother not reporting back into care within two weeks after the calculated delivery date; (3) a newborn not starting nevirapine (NVP) prophylaxis within three days post-partum; (4) a newborn not starting CTX prophylaxis within two weeks after the six weeks post-partum-scheduled treatment initiation date; and (5) a newborn not receiving HIV testing through DNA-PCR within two weeks after the six weeks post-partum-scheduled testing date.

Patients who agreed to be traced through calls to their cell phones were called by a nurse and asked to return to their health facility as soon as possible. If no phone number was provided by the patient or if the call was not successful, a CHW assigned to the patient's residence area conducted a visit to the defaulter's homestead upon request of the nurse in charge. Tracing outcomes were recorded on paper-based forms by the CHW and reported back to the nurse after the home visit. To protect patients’ confidentiality, CHWs did not wear MSF- or HIV/PMTCT-related insignia when conducting the home visit. If the patient did not return into care within one week after the first home visit, a second home visit was carried out by the CHW. Tracing letters were left if no personal contact could be made. If the patient did not report within another week after the second home visit, she was declared LTFU and no further tracing attempts were made. All returning defaulters received at least one counselling session and were channelled back into care.

### Data sources and analysis

Based on patient records, we compared retention in care and perinatal HIV transmission among patients receiving PMTCT services before and after the introduction of CHW-DT in the project in April 2012. The cascade steps under scrutiny were delivery, infant NVP initiation, infant CTX initiation, infant HIV testing and HIV result collection.

During the programme implementation, data from all enrolled PMTCT patients were entered into an MS Excel^®^-based database on an ongoing basis. Clinical information was recorded on paper forms at health facility level by nurses during patient consultations and subsequently entered into the project database together with the corresponding tracing outcome information. Records of all HIV-positive pregnant mothers of all ages and their infants that were newly enrolled into the PMTCT programme at one of the fourteen MSF-supported health facilities in Tsholotsho district between February 2010 and March 2013 were deemed eligible for inclusion into this analysis. Follow-up started with enrolment during ANC and ended with infant HIV testing at six weeks post-partum. The database was censored in July 2013 at the end of the last enrolled patient's follow-up period.

Retention was defined as being alive and in care. For this, patients must have received care within the aforementioned recommended time frames of the respective cascade step or, if she were a defaulter, returned into care after being successfully traced. Defaulting patients that returned into care within two weeks as per tracing algorithm were considered retained, otherwise they were declared LTFU for this analysis. Patients LTFU, transferred out, opted out, or confirmed dead were considered as cases of attrition. This mutually exclusive categorization of retention and attrition followed outcome classification in other defaulter tracing studies [47, 57, 85]. Perinatal transmission was defined as infants testing positive for HIV through DNA-PCR testing at six weeks post-partum.

Age, immunological status, clinical status, antiretroviral (ARV) regimen and treatment area were available baseline characteristics of pregnant women. Age was defined as maternal age at time of enrolment and categorized into five levels. CD4 cell count was taken as proxy for the immunological status of the mother at time of enrolment and categorized into three levels based on commonly used thresholds for clinical decision making. WHO staging served to determine the clinical status of the mother at time of enrolment and was kept as a categorical variable based on its four stages. The fact that eligible women were initiated on lifelong ART for their own health, whereas non-eligible women received Option A for prophylaxis was captured in a binary variable. The different treatment sites were coded binary by grouping together the 12 rural facilities and the two semi-rural facilities.

To describe the overall effects of CHW-DT, we calculated percentages of cumulative retention in care before and after the introduction of CHW-DT among patients who were successfully retained at all cascade steps. Using binomial regression analysis to calculate covariate-adjusted rate ratios (aRR), including 95% confidence intervals (95% CI), we also estimated the specific effects of the CHW-DT intervention on retention at each cascade step separately irrespective of the patients’ successful retention at previous steps, and on perinatal HIV transmission at six weeks post-partum. All available covariates were included as *a priori* risk factors in the models. Software package STATA v. 11^®^ (StatCorp, Texas) was used for this analysis.

### Ethics

Only existing data collected during routine project activities under a Memorandum of Understanding between MoHCW and MSF were used. Data were anonymized and aggregated; hence, the issue of informed consent did not apply. This study met the criteria of the MSF Ethics Review Board (Geneva, Switzerland) for exemption from full ethics review. Exemption from ethics review was also granted by the Medical Research Council of Zimbabwe.

## Results

The total database included 1953 records between February 2010 and March 2013. Six duplicates, 64 entries with impossible or implausible values, and five entries with dates outside the study period were identified and removed, leaving 1878 patient records for analysis. Among these, median maternal age was 27 years (inter-quartile range [IQR] 23 to 32), median CD4 count at enrolment was 394 cells/µL^3^ (IQR 257 to 563), and the number of women classified as having WHO Stage 1 disease was 853 (45.4%). The majority of women (62.2%) originated from rural areas. A total of 998 (53.1%) women were initiated on ART for their own health, the remaining on Option A for prophylaxis (Table 1).

**Table 1:** Patient baseline characteristics

	Before intervention (*N*=1278)		After intervention (*N*=600)			Total (*N*=1878)	
				
	*n* a	%^b^	*n* a	%^b^	*P* c	*n* a	%^b^
CD4 cell count (cells/µL)							
<350	400	31.3	224	37.3	0.066	624	33.2
350 to 499	209	16.3	159	26.5		368	19.6
≥500	336	26.3	137	22.8		473	25.2
Missing	333	26.1	80	13.4		413	22.0
WHO clinical stage							
1	549	43.0	304	50.7	0.971	853	45.4
2	266	20.8	137	22.8		403	21.5
3	249	19.5	139	23.2		388	20.7
4	3	0.2	3	0.5		6	0.3
Missing	211	16.5	17	2.8		228	12.1
Age (years)							
<20	115	9.00	67	11.2	0.525	182	9.7
20 to 24	330	25.8	158	26.3		488	26.0
25 to 29	388	30.3	161	26.8		549	29.2
30 to 34	241	18.9	120	20.0		361	19.2
>34	199	15.6	93	15.5		292	15.6
Missing	5	0.4	1	0.2		6	0.3
ARV regimen							
Treatment	638	49.9	360	60.0	<0.001	998	53.1
Prophylaxis^d^	623	48.8	205	34.2		828	44.1
Missing	17	1.3	35	5.8		52	2.8
Treatment area							
Rural	762	59.6	406	67.3	0.003	1168	62.2
Semi-rural	516	40.4	194	32.3		710	37.8

a Absolute number of column total;

b percentage of column total;

c chi-square test;

d WHO PMTCT Option A.

Clinical and demographic characteristics at enrolment among HIV-positive pregnant women, enrolled before and after the introduction of CHW-DT in April 2012 into the MSF Tsholotsho PMTCT programme between February 2010 and March 2013. ARV: antiretroviral; CD4: cluster of differentiation Type 4 lymphocyte; CHW-DT: community health worker–based defaulter tracing; MSF: Médecins Sans Frontières; PMTCT: prevention of mother-to-child transmission.

A total of 600 (32.0%) women were enrolled after the introduction of CHW-DT in April 2012. CD4 cell count, WHO staging and age distribution did not vary significantly before and after the introduction. However, women enrolled after the CHW-DT introduction tended to be more frequently on treatment for their own health and originate more often from rural than from semi-rural areas (Table 1). The median time point of enrolment was 14.3 weeks (IQR 8.7 to 19.9) and 15.2 weeks (IQR 10.4 to 21.1), respectively, prior to delivery before and after the start of the intervention.

**Figure 1:**
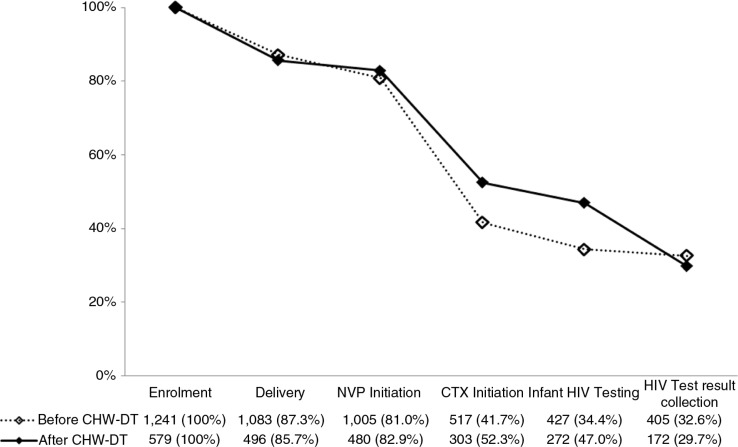
Cumulative retention before and after the intervention. **Cumulative retention in care along the PMTCT cascade among HIV-positive pregnant women and their newborns with complete retention at all previous steps, enrolled before and after the introduction of CHW-DT in April 2012 into the MSF Tsholotsho PMTCT programme between February 2010 and March 2013. CHW-DT: community health worker–based defaulter tracing; CTX: cotrimoxazole; HIV: human immunodeficiency virus; MSF: Médecins Sans Frontières; NVP: nevirapine; PMTCT: prevention of mother-to-child transmission**.

Of the 1878 women in the programme, 1820 (96.1%) were enrolled before giving birth. Before the introduction of CHW-DT, cumulative retention with provision of care within all recommended time periods was 1083 (87.3%) until delivery; 1005 (81.0%) until infant NVP initiation; 517 (41.7%) until infant CTX initiation; 427 (34.4%) until infant HIV testing; and 405 (32.6%) until HIV test result collection. After the introduction of CHW-DT, cumulative retention was 496 (85.7%) until delivery; 480 (82.9%) until infant NVP initiation; 303 (52.3%) until infant CTX initiation; 272 (47.0%) until infant HIV testing; and 172 (29.7%) until HIV test result collection (Figure 1). A stratified analysis showed a similar pattern across treatment sites (Supplementary file 1). A total of 11 patients (1.9%) among those fully retained tested positive for HIV at six weeks post-partum.

After adjusting for CD4 cell count, WHO clinical stage, age, ARV regimen and treatment area (see Table 1), retention at each cascade step separately irrespective of successful retention at previous steps after CHW-DT introduction in April 2012 compared with retention before that date was 85.7 and 84.3% (aRR 1.01, 95% CI 0.96 to 1.07, *p*=0.730) at delivery; 96.6 and 92.0% (aRR 1.35, 95% CI 1.28 to 1.42, *p*<0.001) at infant NVP initiation; 62.0 and 49.1% (aRR 1.78, 95% CI 1.58 to 2.01, *p*<0.001) at infant CTX initiation; and 94.7 and 73.7% (aRR 2.54, 95% CI 2.20 to 2.93, *p*<0.001) at infant HIV testing (Table 2). Correspondingly, perinatal HIV transmission at six weeks among all enrolled patients who received their HIV test result irrespective of retention success was 3.64 and 1.83% (aRR 0.72, 95% CI 0.27 to 1.94, *p*=0.504) (Table 3). There was no consistent pattern in the association between covariates and outcomes (Supplementary files 2 and 3).

**Table 2:** Effects of the intervention on retention

	Patients total		Patients retained							
								
	*n* a	%^b^	*n* a	%^c^	cRR	(95% CI)	*P* e	aRR^d^	(95% CI)	*P* e
At delivery										
Before CHW-DT introduction	1008	63.5	850	84.3	1	(0.97 to 1.06)	0.470	1	(0.96 to 1.06)	0.730
After CHW-DT introduction	579	36.5	496	85.7	1.02			1.01		
At NVP initiation										
Before CHW-DT introduction	850	53.9	782	92.0	1	(1.02 to 1.08)	<0.001	1	(1.28 to 1.42)	<0.001
After CHW-DT introduction	728	46.1	703	96.6	1.05			1.35		
At CTX initiation										
Before CHW-DT introduction	686	49.4	337	49.1	1	(1.15 to 1.39)	<0.001	1	(1.58 to 2.01)	<0.001
After CHW-DT introduction	703	50.6	436	62.0	1.26			1.78		
At infant HIV testing										
Before CHW-DT introduction	346	42.4	255	73.7	1	(1.20 to 1.37)	<0.001	1	(2.20 to 2.93)	<0.001
After CHW-DT introduction	469	57.6	444	94.7	1.28			2.54		

a Absolute number of column total excluding observations with missing data and with the start of the intervention occurring in between the preceding cascade step and the cascade step under comparison;

b percentage of column total;

c percentage of row total;

d adjusted for CD4 count, WHO clinical stage, age, ARV regimen and treatment area;

e likelihood ratio test.

Association between the introduction of CHW-DT in April 2012 and retention in care among HIV-positive pregnant women and their newborns irrespective of completeness of retention at previous steps, enrolled into the MSF Tsholotsho PMTCT programme between February 2010 and March 2013. aRR: adjusted risk ratio; ARV: antiretroviral; CD4: cluster of differentiation Type 4 lymphocyte; CHW-DT: community health worker–based defaulter tracing; cRR: crude risk ratio; CTX: cotrimoxazole; IQR: inter-quartile range; MSF: Médecins Sans Frontières; N: number of patients; NVP: nevirapine; PMTCT: prevention of mother-to-child transmission; 95% CI: 95% confidence interval.

**Table 3:** Effects of the intervention on perinatal HIV transmission

	HIV tests total		HIV tests positive							
								
	*n* a	%^a^	*n* a	%^c^	cRR	(95% CI)	*P* e	aRR^d^	(95% CI)	*P* e
Before CHW-DT introduction	604	57.97	22	3.64	1	(0.23 to 1.12)	0.070	1	(0.27 to 1.96)	0.504
After CHW-DT introduction	438	42.03	8	1.83	0.50			0.72		

a Absolute number of column total excluding observations with missing data and with the start of the intervention occurring at any time during follow-up;

b percentage of column total;

c percentage of row total;

d adjusted for CD4 count, WHO stage, age, ARV regimen and treatment area;

e likelihood ratio test.

Association between the introduction of CHW-DT in April 2012 and perinatal HIV transmission among HIV-positive pregnant women and their newborns irrespective of completeness of retention along the treatment cascade, enrolled into the MSF Tsholotsho PMTCT programme between February 2010 and March 2013. aRR: adjusted rate ratio; ARV: antiretroviral; CHW-DT: community health worker–based defaulter tracing; cRR: crude rate ratio; MSF: Médecins Sans Frontières; N: number of patients; PMTCT: prevention of mother-to-child transmission; 95% CI: 95% confidence interval.

## Discussion

This was the first comprehensive assessment of the effects of CHW-DT on patient care along the perinatal PMTCT cascade in a resource-limited setting. Both before and after the introduction of the CHW-DT intervention, retention was high during pregnancy but decreased sharply after delivery (Figure 1). Although rates for retention increased moderately at post-natal cascade steps after the intervention (Table 2), the proportion of patients successfully retained in care throughout the full treatment cascade remained low overall (Figure 1). In addition, we found no significant reduction in vertical transmission after CHW-DT introduction (Table 3).

Cumulative attrition of about 30% during ANC, 50% at delivery, 70% at four months post-partum, and over 80% at six months post-partum have been reported in different types of studies, including programme implementation studies [27, 41, 45, 86–92]. This picture has been confirmed by a recent meta-analysis that found pooled retention levels of 49% at delivery and 34% at three months post-partum in SSA countries [48]. In our analysis, more than 85% of women were retained in care at time of delivery (Figure 1), which is remarkably higher than findings from a meta-analysis of six PMTCT projects in SSA reporting 49% retention (95% CI 39.6 to 60.9) at time of delivery [48]. The sharp decrease in retention post-partum as observed in our study has also been described elsewhere [27, 41, 45, 86–92]. We found that only 34.4% of mother–infant pairs remained fully retained throughout the cascade until infant HIV testing at six weeks post-partum before introducing CHW-DT (Figure 1), which matches the 34% retention (95% CI 27.6 to 41.5) at three months post-partum found from pooled analysis of 11 PMTCT programmes in SSA [48]. The increased retention after CHW-DT introduction in our study suggests that CHWs could have an added value during the post-partum phase (Figure 1). It does, however, also show that the PMTCT cascade kept leaking at a major scale, particularly considering that this increase disappeared at the subsequent test result collection (Figure 1), which is a crucial step for early ART initiation in HIV-infected infants.

This overall pattern was true for all 14 included sites. Even though most of the 12 rural sites had only small numbers of patients enrolled, which makes stratified findings susceptible to random fluctuation, we found considerable homogeneity of the general trend across sites (Supplementary file 1).

It has been shown that successful retention at all steps throughout the PMTCT cascade is paramount to effectively reduce vertical transmission [25, 33–37]. The CHW-DT intervention in Tsholotsho failed to increase the share of such patients in the cohort. One explanation could be that patients who adhere to all required steps are a highly self-selected group of patients who are likely to comply with recommendations anyways with or without the intervention thanks to favourable access to health or compliance attitude. For other patients, the CHW-DT intervention model as implemented in Tsholotsho might not have been enough to ensure completeness of retention throughout the cascade.

Among patients with complete retention cascade, however, the rate of vertical transmission was 1.9% in our study. This is remarkably low and resembles outcomes from PMTCT programmes in Europe, where high retention is the norm and quality of care is incomparably higher [9]. It confirms the potential of PMTCT interventions to virtually eliminate vertical HIV transmission in SSA if only retention in care and programme coverage were sufficiently high.

Zimbabwe started implementing WHO Option B+ in December 2013 and many SSA countries have been moving in this direction. This will further increase the number of women starting lifelong ART and will require new strategies to fix the leaking retention cascade [93, 94]. Also, infant testing and paediatric HIV care need to be better linked to improve uptake of early ART initiation in children. Further decentralization and integration of PMTCT services with related non-HIV services such as maternal and child care, and combining infant HIV testing with vaccination services is considered promising [95–97]. Our research showed that CHWs are no guarantee for better programme outcomes. Although they might have an important added value in the implementation of some of these strategies, they are no adequate substitutes for necessary adaptations of national health systems and cannot overcome structural shortfalls in service provision in the long run.

Tsholotsho district is a socio-economically disadvantaged part of Zimbabwe with education and access to health care levels well below the national average [78, 98, 99]. The majority of the population lives in dispersed small communities dotted across the district, which requires pregnant women to travel long distances on bad roads to the thin-stretched rural health centres. The ability for many people, including pregnant women, to avail health services is impeded during important times of the agricultural year such as during planting or harvesting seasons. Thus, it is possible that better programme outcomes could be achieved in more favourable settings. However, even under more conducive circumstances, running an active defaulter tracing intervention requires sustained additional investment on top of routine PMTCT programme expenditures. This might be difficult to achieve for the public health sector in many SSA countries without external partners. However, more important than increased financial resources are improvements in human resources and better management. Using CHWs to trace PMTCT defaulters is a low-tech intervention without the need for expensive infrastructure or technical equipment. Good programme management, organizational skills, commitment, and adherence to standardized procedures go a long way in tracing PMTCT defaulters. However, since we do not have data available about the resources invested in the CHW-DT intervention in Tsholotsho, estimations about the transferability of this intervention to other settings and its scalability beyond the district level remain difficult.

Strengths of this study include that 96% of the nearly 2000 available patient records could be included in the analysis, thereby providing a fairly representative sample of all patients enrolled into care in Tsholotsho during the time under review. The project database was the main tool in the project for managing enrolments and patient follow-up. Therefore, regular database maintenance was an integral part of programme activities, keeping accuracy equally high during both observational periods.

Our study was subject to several limitations. Most importantly, the effects of CHW-DT were assessed using a before versus after comparison approach, which makes this research, as all studies relying on historical control groups, vulnerable to confounding through time-associated factors. We attempted to reduce the potential ramifications of this design limitation by adjusting for the most important demographic and clinical baseline parameters. Also, the same PMTCT treatment recommendations (Option A) were in force throughout the study period in our setting [80, 84], which assured conformity of service provision during the time under observation. However, residual confounding through unaccounted factors such as altering health-seeking behaviour or socio-economic shifts over time cannot be ruled out.

Our research only included those patients that received a positive HIV test during ANC and had no population-based component. The HIV prevalence among all ANC patients in the project was not known. Therefore, no coverage estimates could be made.

We could not conduct more in-depth, site-specific investigations. Therefore, variations in outcomes across the different health centres remain difficult to explain.

Furthermore, we could not conduct a more comprehensive process evaluation about the CHW-DT intervention. This would have required qualitative research about knowledge, attitude and practice of PMTCT among the wider population, acceptance of the intervention among enrolled patients, and satisfaction and sustainability among CHWs.

Finally, our analysis only focused on the perinatal transmission period up to six weeks post-partum. Since HIV can also be transmitted through breast milk, final vertical transmission outcomes can only be established six weeks after weaning [100]. This was not covered in our analysis.

## Conclusions

CHW-DT might have the potential to improve retention during the post-natal phase, when attrition is known to be the highest. The intervention did not, however, increase the proportion of patients successfully retained throughout the treatment cascade, and its effects on reduced vertical transmission were not statistically significant at an alpha level of 0.05. We show that in a rural, resource-limited SSA setting, infants whose mothers were fully retained in care as per recommendations can have the same low vertical transmission levels as observed in resource-rich countries. CHWs alone are no magic bullets to improve programme outcomes, as they can only complement but not substitute necessary improvements in service provision by the regular health systems.

## Supplementary Material

Tracing defaulters in HIV prevention of mother-to-child transmission programmes through community health workers: results from a rural setting in Zimbabwe

Tracing defaulters in HIV prevention of mother-to-child transmission programmes through community health workers: results from a rural setting in Zimbabwe

Tracing defaulters in HIV prevention of mother-to-child transmission programmes through community health workers: results from a rural setting in Zimbabwe
