# Supplementary figures and images for: Tracing defaulters in HIV prevention of mother-to-child transmission programmes through community health workers: results from a rural setting in Zimbabwe
Source: J Int AIDS Soc. 2015 Oct 12;18(1):20022. doi: 10.7448/IAS.18.1.20022 (PMC4604210; doi:10.7448/IAS.18.1.20022)

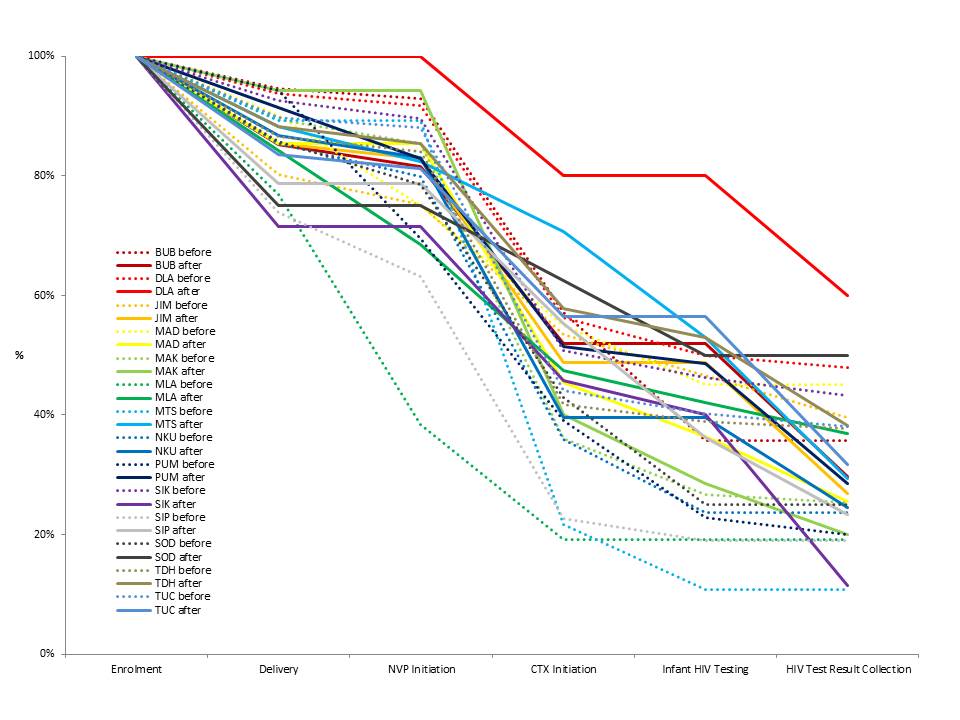

Supplement: Tracing defaulters in HIV prevention of mother-to-child transmission programmes through community health workers: results from a rural setting in Zimbabwe [file JIAS-18-20022-s001.jpg]
