# Supplementary material for: Tracing defaulters in HIV prevention of mother-to-child transmission programmes through community health workers: results from a rural setting in Zimbabwe
Source: J Int AIDS Soc. 2015 Oct 12;18(1):20022. doi: 10.7448/IAS.18.1.20022 (PMC4604210; doi:10.7448/IAS.18.1.20022)
Supplement: Tracing defaulters in HIV prevention of mother-to-child transmission programmes through community health workers: results from a rural setting in Zimbabwe [file JIAS-18-20022-s002.pdf]

	Patients total n[1]	%[2]	Patients retained n[1]	%[3]	cRR	(95%CI)	P-value	aRR[4]	(95%CI)	P-value
**At delivery**										
**CD4 cell count** (cells/µL)										
<350	510	42.2	430	84.3		1			1	
350-499	313	25.9	269	85.9	1.14	(0.76-1.69)	0.143	1.12	(0.70-1.80)	0.040
≥500	387	31.9	344	88.9	1.49	(1.00-2.21)		1.77	(1.10-2.90)	
**WHO clinical stage**										
1	701	51.2	599	85.5		1			1	
2	332	24.3	291	87.7	1.21	(0.82-1.78)	0.588	1.30	(0.85-1.98)	0.430
3	329	24.1	282	85.7	1.02	(0.70-1.48)		1.03	(0.66-1.59)	
4	6	0.4	6	100	-	-		-	-	
**Age** (years)										
<20	155	9.8	125	80.6		1			1	
20-24	429	27.1	365	85.1	1.37	(0.85-2.21)		1.13	(0.61-2.09)	
25-29	464	29.3	385	83.0	1.17	(0.73-1.86)	0.209	0.96	(0.53-1.74)	0.950
30-34	291	18.4	255	87.6	1.70	(1.00-2.89)		1.04	(0.54-1.99)	
>34	243	15.4	211	86.8	1.58	0.92-2.73)		0.95	(0.48-1.84)	
**ARV regimen**										
Treatment	818	53.1	716	87.5		1	0.018		1	1.000
Prophylaxis[5]	723	46.9	602	83.3	0.71	(0.53-0.94)		1.00	(0.63-1.60)	
**Treatment area**										
Rural	978	61.6	825	84.4		1	0.519		1	0.288
Semi-Rural	609	38.4	521	85.6	1.10	(0.83-1.46)		0.83	(0.60-1.16)	
**At NVP initiation**										
**CD4 cell count** (cells/µL)										
<350	517	41.4	493	95.4		1			1	
350-499	314	25.1	302	96.2	1.12	(0.83-1.73)	0.661	1.01	(0.65-1.57)	0.107
≥500	419	33.5	397	94.8	1.29	(0.91-1.82)		1.48	(0.95-2.29)	
**WHO clinical stage**										
1	722	51.4	679	94.0					1	
2	351	25.0	334	95.2	1.19	(0.85-1.68)	0.747	1.35	(0.92-1.97)	0.289
3	327	23.2	306	93.6	0.99	(0.71-1.37)		1.04	(0.69-1.56)	
4	6	0.4	6	100	-	-		-	-	
**Age** (years)										
<20	148	9.4	142	96.0		1			1	
20-24	413	26.3	390	94.4	1.14	(0.73-1.78)		1.23	(0.69-2.21)	
25-29	455	28.9	427	94.0	0.97	(0.63-1.51)	0.515	0.97	(0.55-1.72)	0.633
30-34	312	19.9	288	92.3	1.15	(0.72-1.86)		0.87	(0.47-1.59)	
>34	244	15.5	232	95.1	1.24	(0.75-2.04)		1.10	(0.59-2.07)	
**ARV regimen**										
Treatment	851	55.1	802	94.2		1	0.900		1	0.961
Prophylaxis[5]	694	44.9	653	94.1	0.79	(0.61-1.02)		1.01	(0.66-1.54)	
**Treatment area**										
Rural	975	61.8	897	60.4		1	<0.001		1	0.203
Semi-Rural	603	38.2	588	97.5	1.44	(1.11-1.87)		1.22	(0.90-1.67)	
**At CTX initiation**										
**CD4 cell count** (cells/µL)										
<350	461	41.4	265	57.5		1			1	
350-499	287	25.8	167	58.2	1.03	(0.76-1.39)	0.506	1.11	(0.78-1.59)	0.086
≥500	365	32.8	224	61.4	1.18	(0.89-1.55)		1.44	(1.01-2.05)	
**WHO clinical stage**										
1	633	51.2	353	55.8		1			1	
2	308	24.9	182	59.1	1.15	(0.87-1.51)	0.679	1.17	(0.87-1.58)	0.358
3	290	23.5	164	56.6	1.03	(0.78-1.37)		0.95	(0.68-1.33)	
4	5	0.4	2	40.0	0.53	(0.09-3.19)		0.25	(0.03-2.57)	

**Age** (years)										
<20	133	9.6	69	51.9		1			1	
20-24	358	25.9	185	51.7	0.99	(0.67-1.48)		0.82	(0.52-1.28)	
25-29	403	29.1	233	57.8	1.27	(0.85-1.88)	0.288	1.17	(0.75-1.81)	0.305
30-34	276	20.0	160	58.0	1.27	(0.84-1.94)		0.93	(0.58-1.49)	
>34	213	15.4	124	58.2	1.29	(0.83-2.00)		1.03	(0.63-1.69)	
**ARV regimen**										
Treatment	754	55.4	445	59.0		1	0.011		1	0.067
Prophylaxis[5]	606	44.6	316	52.2	0.76	(0.61-0.94)		0.72	(0.52-1.02)	
**Treatment area**										
Rural	842	60.6	472	56.1		1	0.706		1	0.167
Semi-Rural	547	39.4	301	55.1	0.96	(0.77-1.19)		1.19	(0.52-1.02)	
	**At infant HIV testing**									
**CD4 cell count** (cells/μL)										
<350	280	40.5	237	84.6		1			1	
350-499	174	25.1	155	89.1	1.48	(0.83-2.63)	0.409	1.14	(0.79-1.65)	0.361
≥500	238	34.4	205	86.1	1.13	(0.69-1.84)		1.30	(0.90-1.86)	
**WHO clinical stage**										
1	371	50.1	329	88.7		1			1	
2	196	26.4	163	83.2	0.63	(0.39-1.03)	0.123	1.08	(0.80-1.47)	0.591
3	172	23.2	150	87.2	0.87	(0.50-1.51)		0.99	(0.70-1.40)	
4	2	0.3	1	50.0	0.13	(0.01-2.08)		0.27	(0.03-2.62)	
**Age** (years)										
<20	72	8.9	63	9.0		1			1	
20-24	196	24.1	165	23.6	0.76	(0.34-1.69)		0.79	(0.49-1.27)	
25-29	246	30.3	215	30.8	0.99	(0.45-2.19)	0.752	1.18	(0.75-1.86)	0.243
30-34	166	20.4	139	19.9	0.74	(0.33-1.65)		0.96	(0.58-1.58)	
>34	133	16.3	116	16.6	0.97	(0.41-2.31)		0.90	(0.54-1.50)	
**ARV regimen**										
Treatment	467	58.2	404	86.5		1	0.499		1	0.282
Prophylaxis[5]	336	41.8	285	84.8	0.87	(0.58-1.30)		0.82	(0.58-1.17)	
**Treatment area**										
Rural	494	60.6	398	56.9		1	<0.001		1	<0.001
Semi-Rural	321	39.3	301	43.1	3.63	(2.19-6.01)		1.68	(1.31-2.17)	

Association between patient characteristics at enrolment and retention in care among HIV positive pregnant women and their newborns irrespective of completeness of retention at previous steps, enrolled into the MSF Tsholotsho PMTCT programme between February 2010 and March 2013.

[1] Absolute number of column total excluding observations with missing data and with the start of the intervention occurring in between the preceding cascade step and the cascade step under comparison. [2] Percentage of column total. [3] Percentage of row total. [4] Adjusted for exposure of interest (enrolment before or after the introduction of CHW-DT in April 2012) and all other covariates (CD4 count, WHO clinical stage, age, ARV regimen, treatment area). [5] WHO PMTCT Option A.

aRR: Adjusted Risk Ratio. ARV: Antiretroviral. CD4: Cluster of Differentiation Type 4. CHW-DT: Community Health Worker based Defaulter Tracing. cRR: Crude Risk Ratio. CTX: Cotrimoxazole. HIV: Human Immunodeficiency Virus. MSF: Médecins Sans Frontières. N: Number of patients. NVP: Nevirapine. PMTCT: Prevention of Mother to Child Transmission. WHO: World Health Organization. 95%CI: 95% Confidence Interval.
