# Supplementary material for: Tracing defaulters in HIV prevention of mother-to-child transmission programmes through community health workers: results from a rural setting in Zimbabwe
Source: J Int AIDS Soc. 2015 Oct 12;18(1):20022. doi: 10.7448/IAS.18.1.20022 (PMC4604210; doi:10.7448/IAS.18.1.20022)
Supplement: Tracing defaulters in HIV prevention of mother-to-child transmission programmes through community health workers: results from a rural setting in Zimbabwe [file JIAS-18-20022-s003.pdf]

	Patients total n[1]	%[2]	Patients retained n[1]	%[3]	cRR	(95%CI)	P-value	aRR[4]	(95%CI)	P-value
**CD4 cell count** (cells/µL)										
<350	349	41.3	11	3.2		1			1	
350-499	210	24.8	7	3.3	1.06	(0.40-2.78)	0.155	0.53	(0.11-2.47)	0.096
≥500	287	33.9	3	1.1	0.32	(0.09-1.17)		0.18	(0.03-1.01)	
**WHO clinical stage**										
1	465	50.0	16	3.4		1			1	
2	232	24.9	7	3.0	0.87	(0.35-2.15)	0.7663	0.68	(0.21-2.23)	0.517
3	231	24.8	0	0.0	-	-				
4	3	0.3	0	0.0	-	-				
**Age** (years)										
<20	85	8.2	2	2.4		1			1	
20-24	249	24.0	11	4.4	1.92	(0.42-8.83)		2.24	(0.25-19.97)	
25-29	308	29.7	8	2.6	1.11	(0.23-5.31)	0.485	1.35	(0.14-12.63)	0.890
30-34	217	20.9	4	1.8	0.78	(0.14-4.34)		1.92	(0.19-19.88)	
>34	179	17.2	4	2.2	0.95	(0.17-5.28)		0.51	(0.16- 1.61)	
**ARV regimen**										
Treatment	574	55.9	8	1.4		1	0.002		1	0.114
Prophylaxis[5]	452	44.1	21	4.7	3.45	(1.51-7.86)		3.34	(0.73-15.27)	
**Treatment area**										
Rural	651	62.5	23	3.5		1	0.103		1	0.294
Semi-Rural	391	37.5	7	1.8	0.50	(0.21-1.17)		0.54	(0.17-1.76)	

Association between patient characteristics at enrolment and perinatal HIV transmission among HIV positive pregnant women and their newborns irrespective of completeness of retention at previous steps, enrolled into the MSF Tsholotsho PMTCT programme between February 2010 and March 2013.

[1] Absolute number of column total excluding observations with missing data and with the start of the intervention occurring in between the preceding cascade step and the cascade step under comparison. [2] Percentage of column total. [3] Percentage of row total. [4] Adjusted for exposure of interest (enrolment before or after the introduction of CHW-DT in April 2012) and all other covariates (CD4 count, WHO clinical stage, age, ARV regimen, treatment area). [5] WHO PMTCT Option A.

aRR: Adjusted Risk Ratio. ARV: Antiretroviral. CD4: Cluster of Differentiation Type 4. CHW-DT: Community Health Worker based Defaulter Tracing. cRR: Crude Risk Ratio. MSF: Médecins Sans Frontières. N: Number of patients. PMTCT: Prevention of Mother to Child Transmission. WHO: World Health Organization. 95%CI: 95% Confidence Interval.
